# Academy of Stomatology

**Published:** 1902-12

**Authors:** 

**Affiliations:** Academy of Stomatology


### ACADEMY OF STOMATOLOGY.

A REGULAR meeting of the Academy of Stomatology of Philadelphia was held at its rooms, 1731 Chestnut Street, on the evening of Tuesday, April 22, 1902, the President, Dr. S. B. Luckie, in the chair.

A paper entitled "Stomatitis from a Dental Stand-Point" was read by Dr. David Genese, of Baltimore.

(For Dr. Genese's paper, see page 644.)

DISCUSSION.

*Dr. James Truman.*—I had an opportunity to-day of listening to Dr. Genese talk upon several topics, and to me they were profoundly interesting, especially his method of manipulation of plaster. It was an entire novelty to me, and in order to bring the method before the society I move that the regular order be changed, and that he be requested to proceed with the demonstration he has kindly promised to give.

Motion carried.

*Dr. D. Genese.*—The models which I have here were taken from impressions made with plaster in a way which I will show you, and without inconvenience to the patient. In mixing plaster for this work it is hardly necessary to soil the table.

The method is very simple. I use no water, but a preparation made from the gluten of rice. It is almost a syrup, but without any adhesiveness to it, yet it has a great affinity for the plaster and will take up a very large quantity, and enough is added so that it can be rolled in the hands like a piece of putty. There is no need for haste. When set it has a beautiful smooth surface, as though cast, and no air-bubbles remain in it. If the ordinary plaster is dropped into water, allowed to settle thoroughly, the excess of water poured off, and then a teaspoonful of this gluten is added, it will make the mixture smooth, and it will pour without any air-bubbles. It will remain plastic for some time, and when placed in the mouth and an impression taken it will not run off the tray, nor will the edges break away. It requires a little longer time to set than the ordinary plaster.

I use a preparation here to prevent plaster from adhering. It does not have to be colored. You can pass it over a plaster cast, pour the plaster on, and all that is necessary before separation is to put it in water. In taking a large cast of the face, I would apply some of this non-adhesive compound to the face and then put the plaster mixed in this way over the parts without fear of catching the hair or giving the slightest pain, and without breakage. I can put it all over the head and it will not adhere if the composition has been previously applied. There is no heat evolved in setting, as is the case with the ordinary plaster-and-water mixture. When it begins to set it is a little friable under pressure, and instead of keeping the imprint has a tendency to part a bit, which shows that it should not be further manipulated.

It is very difficult to add plaster to plaster. Plaster models get broken, and you cannot repair them with ordinary plaster. The moment the new plaster comes in contact with the old the moisture is absorbed, and afterwards the added portion will fall away. The glutenized plaster can be added to any part previously wet, and it does not move from its place when hard. If one happen to fracture a model, mix a little plaster as I have suggested, touch the two parts with the material, press them together, and wipe off the excess; then put on a warm stove, and in ten minutes the line of fracture cannot be recognized and the model will not again break in the same place. A suction disk may be made to adhere to a model with it, and it will occupy no appreciable space; yet it will go through vulcanization without detachment of the disk from the model. In vulcanizing with this plaster mixture it will undergo no change, and when a piece is vulcanized it will come away, and one can make another set on the model without any inconvenience.

(Replying to questions.)

The parting medium is a solidified glycerin made by the stearate process, is very simple, and it is now ready for the profession. A small portion is taken from the bottle and spread over the plaster with the finger-tip or a fine brush. It does not injure the model. I do not think salt would unite with the composition. It has a tendency to destroy in part the integrity of the plaster and to make it rotten when water touches it. It will always cause the plaster to absorb moisture. In this preparation moisture dries out, particularly with the aid of heat, and the union of the particles of plaster is exact. It can be subjected to considerable heat without injury. In case of fracture of an impression the pieces do not fall into such small fragments that you cannot pick them up and put them together.

The material is also useful for the reproduction of any works of art by electro-deposition. To copy a bronze figure is a very difficult matter in modelling, but if this is used for the mould and then boiled in paraffin it will take the deposit of copper as nicely as any material. I intended to bring you a figure of a dog that I did.

There is another method of modelling in copper that I do not think is known. The old copper amalgam is a precipitate of copper from a cupric sulphate solution by means of zinc sheets. This is amalgamated under a solution of sulphuric acid, then washed free of acid, dried, and packed into moulds to crystallize. If any of you want to copy a statue, make a mould of it, pack it full of this copper amalgam, let it harden gradually, then heat it in a fire until the globules of mercury appear, and by the time it is heated to a red heat you will have an absolutely pure copper figure, and it is the hardest copper I have ever come across. The operation should be conducted under a chimney draught.

*Dr. James Truman.*—I would like to ask how the gluten is prepared?

*Dr. Genese.*—It is made from rice. The idea accidentally occurred to me while making rice capsules, which are soluble in water. In manipulating the rice I spoiled it, getting a liquid instead of a solid, and this I experimented with. Rice can be made so perfectly hard by manipulation that picture-frames can be made of it; in fact, anything that you care to mould. Professor Neal, of the University of Maryland, ordered a set of pelvic cavities from Tramond, of Paris, which arrived broken to pieces. While working on these, at Dr. Neal's request, I thought of the possibilities in glutenizing plaster. In dental work it is almost indispensable. You will see the value of it in this imprint of the ear, which ordinarily is troublesome to get. I have done three cases of compound fracture of the lower jaw within ten minutes for each, and in two cases out of the three the muscles had drawn the fractured parts out of line.

*The President.*—Discussion of Dr. Genese's paper is now in order. Dr. Kirk, will you open the discussion?

*Dr. E. C. Kirk.*—As the paper was being read I was impressed with the importance of the observation made by Dr. Genese as to the necessity for the correction of constipation in the first case that he reports. I think a great deal of light, in fact, the first real light that was thrown upon the etiology of stomatitis came to us in the shape of a paper read at the Third International Dental Congress by Dr. Loup, of Paris. His paper dealt with the *rôle* of mercury in the production of so-called mercurial stomatitis. Dr. Loup showed very conclusively that there was no such thing as mercurial stomatitis, properly speaking, and that the function of the mercury in all the cases where we have stomatitis is the production of a tissue intoxication, which at certain stages makes the mouth tissues vulnerable to the bacteria, which are its constant inhabitants. He proved that by increasing the dose of mercury a bactericidal effect was produced and the stomatitis relieved.

In Dr. Genese's case we have a set of conditions quite analogous.

In the first place, his case had been treated with morphine for neuralgia to the point where the bodily secretions were completely checked, and during the time this state existed there was undoubtedly an autointoxication from the absorption of intestinal waste products. In addition to that, mercurials having been given as a cholagogue and to evacuate the intestinal contents, there was a distinct poison given to the system, already saturated, so that infection was promoted by the depression of the vitality of the tissues. His treatment of the case was logical. The correct treatment after the evacuation of the intestinal tract by the use of glycerin suppositories is to follow up this medication with salts. He thus reduced the toxæmia and was enabled to take hold of the case, raising the standard of vital resistance to something like normal. It seems to me that this point is a very important one and worthy of consideration.

*Dr. James Truman.*—I do not exactly like the term "stomatitis." We have all sorts of inflammations about the mouth, and nearly all of them go under that general head. The aphthæ of children and thrush have special names. Stomatitis is simply an inflammation of the mouth. There are different diseases that are separated, like leucoplakia, which has its origin in chronic irritation. Most of these cases that Dr. Genese has cited seem to me, as he described them, to be due to a gastro-intestinal disturbance. That agrees with my observation in cases of stomatitis as generally recognized by that name. There is one class of cases, however, of which I am in doubt as to whether it should be placed under that head or not. In these cases there is a partial hyperæmia of the mouth and an intense redness over a portion only of the mucous membrane. Perhaps some of those more familiar with the condition can explain it as due to an intestinal or gastric disturbance. If so, why should it not attack the entire mouth? I have seen a number of these cases in the University of Pennsylvania clinic. Of course, the paper deals with cases rather than general principles. Therefore, I do not propose to take up general principles outside of the paper.

*The President.*—Dr. Genese will close the discussion.

*Dr. Genese.*—Such a wide field is open for research in this condition, which we meet with daily in our practices, that nothing but absolute experience at the side of our patients or in the hospitals will give an idea of the number of the obscure reflexes. I was at the clinic at the University of Maryland and saw a remarkable pathological specimen presented that had baffled both physicians and dentists for a long time, until it was decided, after a thorough examination by both practitioners, that an operation should be performed to remove a cause not clear to ordinary diagnostic means, though the abdominal wall was distended. A cyst was removed from the walls of the stomach containing a tooth that had evidently been there from embryo life and in a fully developed condition. So go on indefinitely the causes met with.

*Dr. E. T. Darby.*—They are not rare.

*Dr. Genese.*—It was the first case I came across, and contained a bicuspid tooth. The derangement of the system of the patient continued for several years. I think inquiry should be made of our patients when they present themselves looking as though unable to endure an operation, and that we should put ourselves on the safe side by declining to operate, in order to avoid shock when the patient is not in a condition to receive the attention given.

*Dr. James Truman.*—I move that the thanks of the Academy be given to Dr. Genese for his very interesting paper and demonstration.

Carried.

*The President.*—Dr. Genese has the thanks of the Academy.

Adjourned.

OTTO E. INGLIS, D.D.S.,
*Editor Academy of Stomatology.*

